# Trans-olecranon fracture posterior dislocation: a novel type of elbow injury

**DOI:** 10.1186/s13018-023-03563-5

**Published:** 2023-03-22

**Authors:** Fulin Tao, Dongsheng Zhou, Wenhao Song

**Affiliations:** 1 Department of Orthopedic Surgery, Shandong Provincial Hospital, Cheeloo College of Medicine, Shandong University, 324 Jingwu Road, Jinan, Shandong China; 2 Department of Orthopedic Surgery, Shandong Provincial Hospital Affiliated to Shandong First Medical University, 324 Jingwu Road, Jinan, Shandong China

**Keywords:** Elbow joint dislocation, Olecranon fracture, Severe elbow instability

## Abstract

**Background:**

Based on our experiences, we found that a kind of elbow injury is characterized by an olecranon fracture accompanied by elbow joint posterior dislocation with the proximal radioulnar joint intact. The aim of this study was to better define this kind of severe elbow instability, which has not been previously reported.

**Methods:**

We retrospectively analyzed all patients with olecranon fractures who were treated at our institution from January 2013 to April 2021. Data on these patient demographics, injury characteristics, preoperative and postoperative imaging, surgical management, and outcomes were recorded and analyzed. We also made the inclusion criteria and exclusion criteria.

**Results:**

A total of 309 patients were diagnosed olecranon fractures in our institution, and ten patients met the inclusion criteria, 9 males and 1 female, with an average age of 40.6 ± 12.7 years (26–68 years). Eight patients (80%) were comminuted, and two were oblique olecranon fracture. Nine patients (90%) suffered coronoid process fractures, eight fractures were type III, and one was type II. Eight patients (80%) suffered radial head fractures, seven fractures were type II, and one was type III. All patients suffered lateral collateral ligament complex injury. All patients underwent surgical management and were followed up on average for 15.8 ± 3.2 months (12–20 months). The motion of the elbow and functional outcome were evaluated with several methods. The mean arc of the elbow movement was 131.6° ± 6.0° (124°–140°), and the mean arc of the forearm rotation was 158.5° ± 17.8° (128°–180°). Nine patients’ functional results according to the Mayo Elbow Performance Score (MEPS) were excellent with a mean score of 96.5 ± 5.3 points (85–100 points), and another was good. The mean score according to the Broberg and Morrey functional rating index was 98.8 ± 2.5 points (92–100 points), nine patients were excellent, and another was good. The mean Disabilities of the Arm, Shoulder, and Hand (DASH) score was 0.75 ± 1.2 points (0–3.3 points).

**Conclusions:**

Trans-olecranon fracture posterior dislocation is a rare injury and has unique characteristics, and it is a kind of complex elbow instability involving the coronoid process and radial head fractures. After bony structure is restored, the repairment of lateral collateral ligament complex is also important to the stability of the elbow joint. Correct understanding of this kind of injury and reasonable treatment plan can achieve good function.

## Introduction

**Fig. 1:**
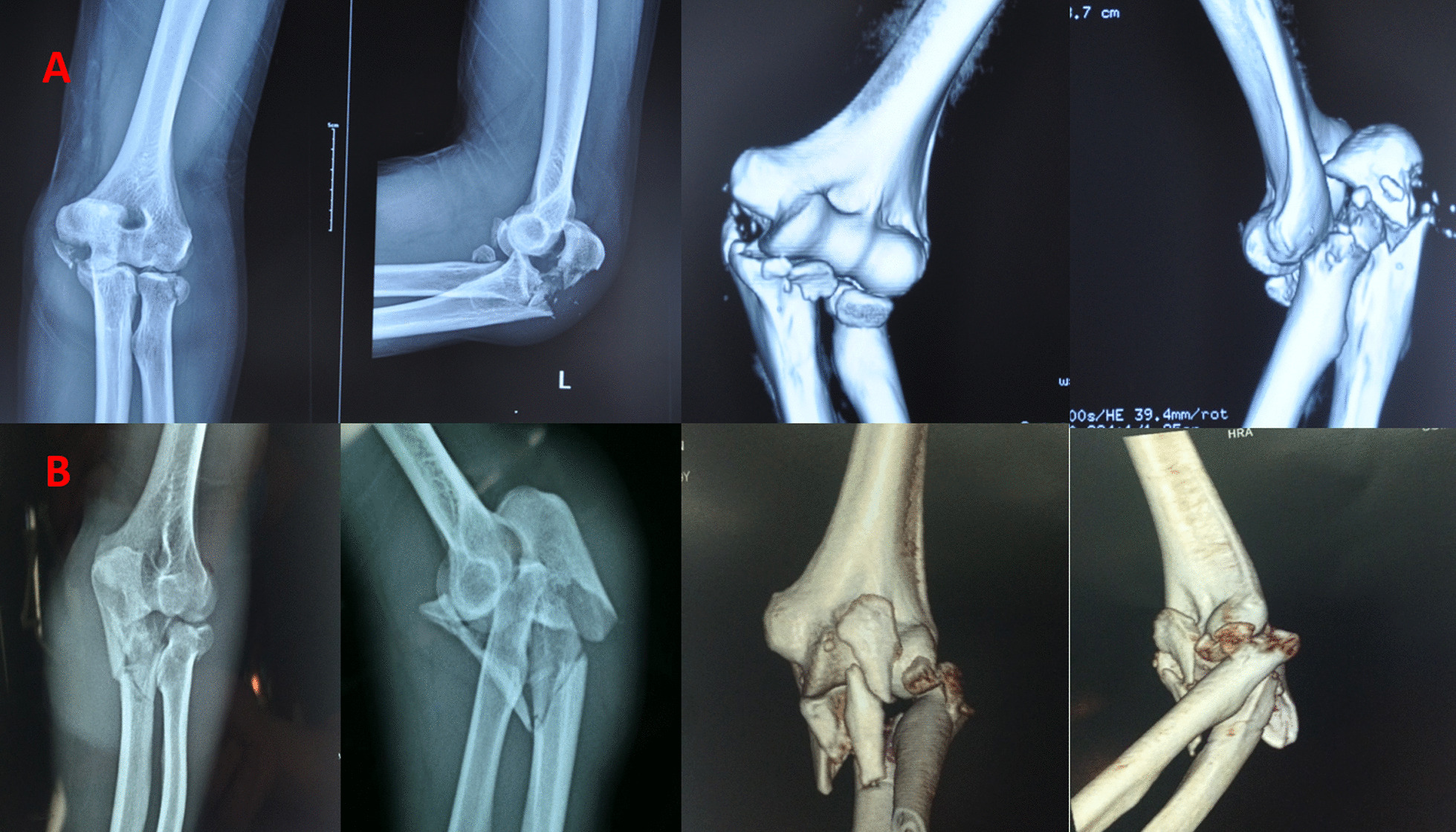
Trans-olecranon fracture posterior dislocation is different from posterior Monteggia fractures. **A** Trans-olecranon fracture posterior dislocation: olecranon fractures, posterior dislocation of the elbow, and stable proximal radioulnar joint. **B** Posterior Monteggia fractures (type Bado IIA): olecranon fractures, posterior dislocation of the radial head, and disruption of the proximal radioulnar joint

Olecranon fractures with elbow dislocation are rare, which can cause serious elbow instability [1]. Studies of olecranon fracture dislocations of the elbow were rare due to its low incidence; therefore, there is no uniform standard for the diagnosis, classification, and treatment. At present, it is considered that there are two kinds of such injuries: One is olecranon fractures with anterior dislocation, and the other is olecranon fractures with posterior dislocation [2]. There are many articles about olecranon fractures with anterior dislocation [3, 4]. In 1974, Biga [3] defined olecranon fractures with anterior dislocation as trans-olecranon fracture dislocation, and many scholars clearly pointed out that this injury is the result of fracture through the olecranon, while the proximal radioulnar joint remains stable [3, 4]. This characteristic distinguishes it from the anterior Monteggia fractures in which the proximal radioulnar joint is disrupted [2]. Also, almost all studies considered that olecranon fractures with posterior dislocation were a type of posterior Monteggia fracture (type Bado IIA) [5, 6], which is associated with disruption of the proximal radioulnar joint and possible injury of the lateral ulnar collateral ligament. However, based on our experiences, we found that not all olecranon fractures with posterior dislocation were posterior Monteggia fractures. There exist a kind of rare injury which is characterized by: posterior dislocation of the elbow joint, olecranon fracture, and stable proximal radioulnar joint (Fig. 1). This type of injures has never been reported. Therefore, the aim of this study was to better define and characterize this injury and try to provide additional data to indicate the rule of treatment.

## Methods and patients

We retrospectively analyzed all patients with olecranon fractures who were treated at our institution from January 2013 to April 2021. Informed consent was obtained from all patients.

### Inclusion and exclusion criteria

We analyzed the demographic data, preoperative and postoperative imaging (X-ray, computerized tomography), and made the inclusion criteria: (1) older than 16 years, (2) olecranon fracture, (3) posterior dislocation of the elbow joint, (4) stable proximal radioulnar joint, and (5) clinical and radiological records were complete. Exclusion criteria were established as: (1) patients who had a previous elbow trauma history, (2) follow-up time less than 1 years, (3) incomplete clinical and/or radiological data.

### Collected data

We collected the following data for each patient: gender, age, fracture type, cause of injury, associated injuries, open fracture (yes or no), operative approach, fracture fixation, ligamentous injury (yes or no) and repair methods, follow-up time, complications, Broberg and Morrey score [7], Mayo Elbow Performance Score (MEPS) [8], and Disabilities of the Arm, Shoulder, and Hand (DASH) score [9]. Several classifications exist for elbow fractures, we used the Mason system [10] to evaluate radial head fractures, and the Regan–Morrey system [11] was used to classify coronoid process fractures.

### Functional evaluated system

At the last follow-up, the functional status of the elbow was evaluated using the above assessment system. The rating system of Broberg and Morrey is a 100-point system, which consists of four sections: motion, strength, stability, and pain. The MEPS assessment system includes pain, ulnar–humeral motion, stability, and the ability to perform activities of daily life, with higher scores indicating better function. We used DASH, a 30-item, self-reported instrument to evaluate the physical function of the upper limb. The flexion and extension of the elbow as well as the range of pronation and supination were measured.

## Results

### Demographic data

During the study period, 309 patients were diagnosed olecranon fractures in our institution, and 10 patients met the inclusion criteria, 9 males and 1 female, in the age range 26–68 years, average 40.6 ± 12.7 years. Eight patients suffered a fracture in the dominant arm, and one patient suffered an open fracture (Gustilo I). The main cause of injuries was falling, and there were 5 patients. Three patients suffered falling from a height. Two patients suffered from a traffic injury. (One was a motorcycle–car accident, and the other was a pedestrian–car accident.) Six patients had only an elbow injury, one suffered a lumbar vertebrae fracture, one patient suffered a lumbar vertebrae fracture and a pelvic fracture, one patient suffered a craniocerebral injury, and one patient had a nasal bone fracture. The details are shown in Table 1.

**Table 1:** Demographic information

No	Gender	Age	Injury mechanism	Associated injuries	Open fracture	FITO (days)	Dominant arm
1	M	42	Fall	None	No	5	Yes
2	M	26	Fall	None	No	8	Yes
3	M	51	Fall from height	None	No	12	Yes
4	M	46	Fall from height	LF, PF	No	6	Yes
5	M	26	Motor vehicle injury	Brain injury	No	7	Yes
6	M	34	Fall	None	No	5	Yes
7	M	39	Traffic accident	LF	No	7	No
8	M	31	Fall	None	No	3	Yes
9	M	43	Fall from height	NF	Yes	14	Yes
10	F	68	Fall	None	No	5	No

*FITO* from injury to operation, *LF* lumbar vertebrae fracture, *PF* pelvic fracture, *NF* nasal bone fracture

### Radiographic classification

All patients underwent an anterior–posterior and lateral X-ray examination of the elbow, and a computerized tomography (CT) examination was also necessary. Based on the results of X-ray and CT, we judged and classified the fracture types. Two patients suffered an oblique fracture of the olecranon, and the rest suffered a comminuted fracture of the olecranon. Nine patients suffered a coronoid process fracture, eight patients were classified as type III, and one patient was classified as type II based on the Regan–Morrey system. Eight patients suffered a radial head fracture, seven patients were classified as type II, and one patient was classified as type III based on the Mason system.

### Surgical technique

**Fig. 2:**
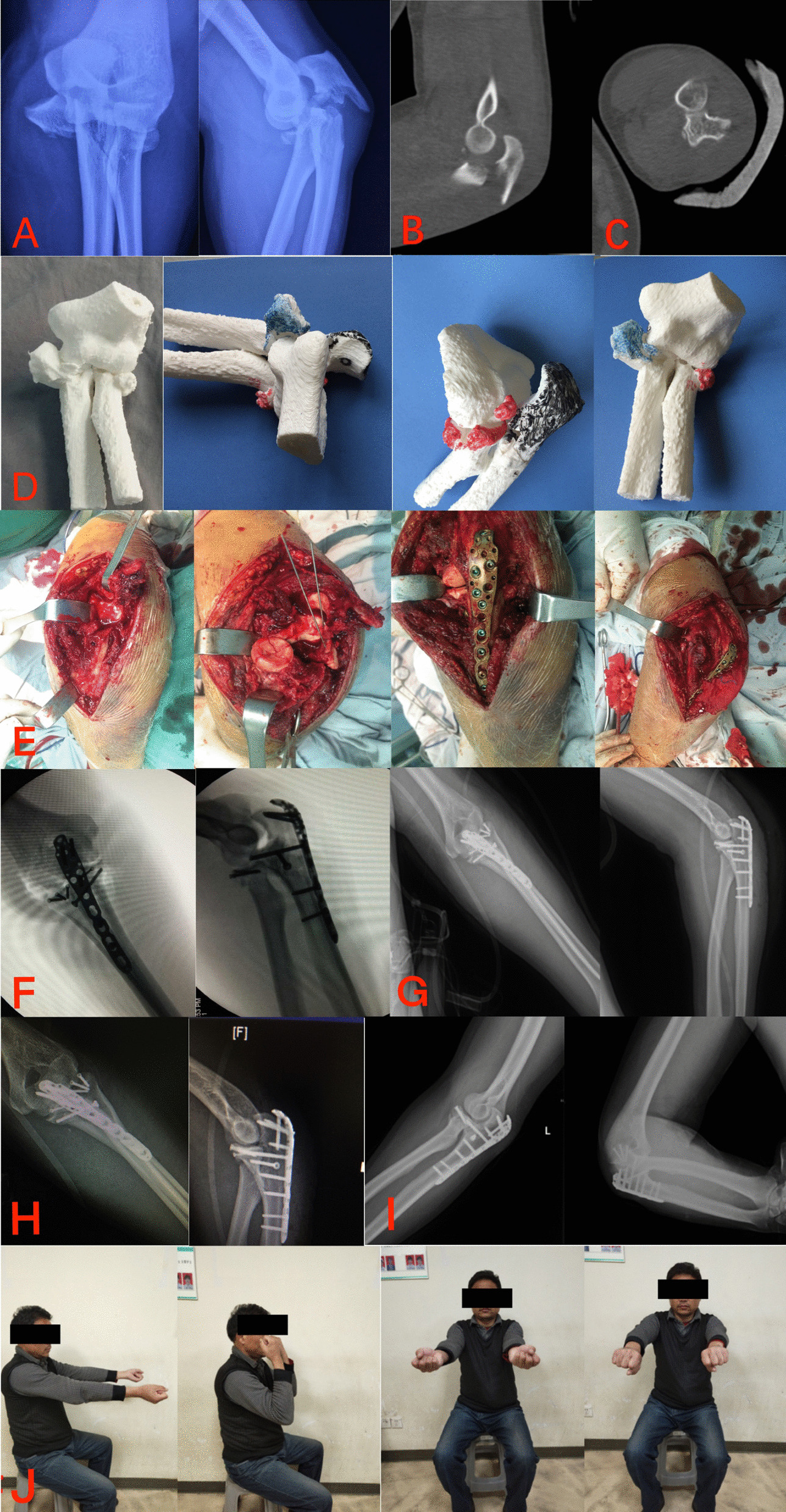
Male, 34 years old, falling caused left elbow injury. **A** Preoperative X-rays. **B** CT scan showed olecranon fracture and elbow posterior dislocation. **C** CT scan showed proximal radioulnar joint stable. **D** For better surgical guidance, we printed a 3D model, the olecranon fracture fragment is shown in black, the radial head fracture fragment in orange, and the coronoid process fracture fragment in blue. **E** Posterior midline approach was used, and the proximal olecranon fragment was identified and turned over. Coronoid process fragment and radial head fragment can be visualized due to the absence of olecranon obstruction. After the reduction and fixation of coronoid process and radius head, the olecranon fragments were also reduced and fixed. Finally, the lateral collateral ligament complex was examined and repaired if it was ruptured. **F** Intraoperative fluoroscopy. **G** Postoperative X-rays. **H** X-rays after surgery 6 months. **I** X-rays after surgery 1 year. **J** The functional assessment at last follow-up

All patients underwent operation within two weeks of injury, and the duration of surgery ranged from 3 to 14 days, with a mean of 7.3 ± 3.4 days. Two patients received general anesthesia, and the others received brachial plexus anesthesia. Nine patients received a posterior midline approach, and one patient received a posterior midline approach and an anterior approach, with the ulnar nerve usually unidentified. After the proximal olecranon fragment was identified and turned over, the coronoid process and radius head could be visualized. Generally, the coronoid process fragment was large and could be reduced and fixed with screws through the posterior midline approach. One patient experienced a small fragment of coronoid process, and the anterior approach was used to expose, reduce, and fix the small fragment. Meanwhile, the radial head can be visualized through a posterior midline approach, which reduces the fragment and allows for easy fixation due to the absence of olecranon obstruction. Restoration of ulnar anatomy is the most important step for severe elbow injury. After the reduction and fixation of coronoid process and radius head, the olecranon fragments were also reduced and fixed. Finally, the lateral collateral ligament complex was examined and repaired if it was ruptured. In this paper, we report eight patients with radial head fractures, all of which were fixed with screws. Nine patients had coronoid process fractures, two patients were fixed with K-wire, and the other patients were fixed with screws. All patients suffered olecranon fractures, two patients were fixed with a tension band, and the other olecranon fractures were fixed with plates. All patients suffered a compound injury of the lateral collateral ligament, which was repaired by bone drilling and suturing in four patients and by anchors in six patients. Patients achieved elbow stability after the repair of the bone and ligament injuries. The type of fracture and fixation method are detailed in Table 2. The surgical procedures are shown in Fig. 2.

**Table 2:** Type of fracture and fixation, treatment

No	Olecranon fracture		Coronoid process fracture		Radial head fracture		LCL complex injury	
	Type of fracture	Fixation method	Type of fracture	Fixation method	Type of fracture	Fixation method	Yes or no	Repair method
1	Comminuted	ALP	II	K-wire	II	Screws	Yes	Anchors
2	Comminuted	ALP	III	Screws	II	Screws	Yes	BDS
3	Comminuted	ALP	III	Screws	II	Screws	Yes	BDS
4	Comminuted	TB	III	Screws	II	Screws	Yes	Anchors
5	Comminuted	ALP	III	Screws	II	Screws	Yes	Anchors
6	Oblique	ALP	No fracture	–	No fracture	–	Yes	Anchors
7	Comminuted	ALP	III	Screws	No fracture	–	Yes	Anchors
8	Comminuted	ALP	III	Screws	II	Screws	Yes	Anchors
9	Comminuted	ALP	III	Screws	III	Screws	Yes	BDS
10	Oblique	TB	III	K-wire	II	Screws	Yes	BDS

*ALP* anatomical locking plate, *TB* tension bands wire, *LCL* lateral collateral ligament, *BDS* bone drilling and suturing

Postoperative rehabilitation exercise is also important. The exercise program is summarized as follows: All patients did not use plaster fixation and began functional exercise on the fifth day after surgery. Pain relievers can be applied prior to exercise to reduce pain. Patients were asked to gradually bend the elbow and then gradually straightened it again. We asked patients to perform similar functional exercises twice a day, once in the morning and once in the evening.

### Outcome measures

All patients were asked to bend and stretch the elbow 1–2 times a day after the operation. All patients were followed up, and follow-up time was from 12 to 20 months, average 15.8 ± 3.2 months. All patients had no complications.

All patients received functional evaluation at the last follow-up. The active range of motion and functional status of the elbow were evaluated. The mean arc of the elbow movement was 131.6° ± 6.0 (124–140°), with a mean flexion of 135.8° ± 5.1° (range 127–140°) and a loss of extension of 4.2° (range 0–12°). The mean arc of the forearm rotation was 158.5° ± 17.8° (range 128–180°). The means of pronation and supination of the forearm were 79.6° ± 9.0 (68–90°) and 78.9° ± 11.0 (60–90°), respectively. Nine patients’ functional result according to MEPS was excellent, and only one patient was good, with a mean score of 96.5 ± 5.3 points (85–100 points). The mean score according to the Broberg and Morrey functional rating index was 98.8 ± 2.5 points (92–100 points), only one patient was good, and the rest were excellent. The mean DASH score was 0.75 ± 1.2 points (0–3.3 points). The outcomes of follow-up are shown in Table 3. We chose one case as the classical cases shown in Fig. 2.

**Table 3:** Active range of motion and functional status of the elbow

No.	Follow-up (months)	active range of motion		functional status of the elbow		DASH
		F/E	P/S	MEPS	Broberg and Morrey index	
1	59	130–0–0°	80–0–67°	100, excellent	98.7, excellent	0
2	42	140–0–0°	90–0–90°	100, excellent	100, excellent	0
3	60	128–0–0°	68–0–68°	95, excellent	98.4, excellent	0.83
4	38	140–0–0°	85–0–90°	100, excellent	100, excellent	0
5	62	137–10–0°	73–0–87°	95, excellent	100, excellent	0.83
6	20	140–12–0°	90–0–74°	100, excellent	100, excellent	0
7	18	129–0–0°	90–0–90°	100, excellent	98.8, excellent	0
8	22	134–10–0°	80–0–83°	100, excellent	99.8, excellent	0
9	85	140–0–0°	72–0–80°	90, excellent	100, excellent	2.50
10	104	140–10–0°	68–0–60°	85, good	92, good	3.33

*F* Flexion, *E* Extension, *P* Pronation, *S* Supination

MEPS: Mayo Elbow Performance Score, Score: 90–100 points: excellent, 75–89 points: good, 60–74 points: fair, < 60 points: poor

Broberg and Morrey functional rating index, Score: 95–100 points: excellent, 80–94 points: good, 60–79 points: fair, < 60 points: poor

DASH: Disabilities of the Arm, Shoulder, and Hand Outcome Measure

## Discussion

### It is different from posterior Monteggia fractures

Olecranon fracture dislocations are fractures of the olecranon associated with elbow instability, and the forearm may dislocate in anterior (trans-olecranon) or posterior directions. Trans-olecranon fracture dislocation was first reported by Biga and Thomine [3]. It was defined as an olecranon fracture associated with anterior dislocation of the forearm, with the proximal ulnoradial joint being stable [3, 4]. However, there are few reports about olecranon fractures combined with forearm posterior dislocation. Most scholars believed that this injury is a kind of Monteggia fracture [5, 6]. In 1914, Monteggia described this injury of a proximal ulnar fracture associated with anterior dislocation of the radial head. In 1967, the Monteggia fracture was classified based on the location of the ulna fracture and direction of the dislocation of the radial head, and the presence or absence of a concomitant proximal radius fracture [12]. Posterior Monteggia fractures (Bado type II) were subdivided by Jupiter and colleagues based on the ulnar fracture in relation to the coronoid [13]. Based on classified systems, an olecranon fracture associated with a forearm posterior dislocation is Bado type IIA which defined proximal ulna fractures combined with radial head posterior dislocation. However, we found that part of olecranon fractures with a forearm posterior dislocation were not Monteggia fractures because the proximal ulnoradial joint was stable. To distinguish it from other injuries, we named this injury as the trans-olecranon fracture posterior dislocation.

### The characteristics of this injuries

Many scholars summarized three main patterns of complex elbow instability, which include posterolateral (terrible triad), varus posteromedial (anteromedial coronoid fracture with lateral collateral ligament complex disruption), and trans-olecranon fracture dislocations [14]. Trans-olecranon fracture posterior dislocation is very different from these three kinds of injuries. From this study, we summarized that the clinical characteristics are: (1) posterior dislocation of the elbow and stable proximal radioulnar joint, (2) olecranon fracture is mostly a comminuted fracture, (3) most patients had coronoid process fractures, most of which were Regan–Morrey type III, with large fragments, (4) most patients also had radial head fractures, most of which were Mason type II, and (5) the lateral collateral ligament complex was disrupted.

### This is a kind of complex elbow instability

Just as in other complex elbow injuries, trans-olecranon fracture posterior dislocation can also cause severe complex elbow instability, which means that patients suffered bone and ligament injuries. Bones, ligaments, and muscle–tendon units maintain elbow stability [15–18]. As a whole, the coronoid process plays an important role in maintaining the stability of the elbow because of the attachments of the ulnar collateral ligaments which can provide varus, valgus, posteromedial, and posterolateral stability of the elbow [2]. The coronoid process is also an important structure that prevents forearm posterior dislocation [5, 19]. Therefore, trans-olecranon fracture posterior dislocation is always combined with coronoid fractures. In this study, 90% of patients suffered coronoid process fractures, and most of them were Regan–Morrey type III. The radial head serves as a secondary stabilizer to valgus forces. Radial head fractures may result in a kinematic change of the elbow, especially in the case of a rupture of the collateral ligament [20]. In this study, we found 80% of cases suffered radial head fractures. Most of them were classified as Mason type II. The medial and lateral ligament structure also plays an important role in the stability of the elbow joint [1, 20–23]. The lateral collateral ligament complex is composed of the radial collateral ligament, lateral ulnar collateral ligament, and the annular ligament. The lateral ulnar collateral ligament is considered the primary stabilizer of the elbow to varus and posterolateral rotatory stress. In our study, we found that all patients’ lateral collateral ligament complexes were disrupted. Dislocation still occurs in most patients after the bony structure is restored, which means the ligaments are still broken. After we repaired the ligaments using anchors or sutures, the elbow stability was restored.

### The mechanism of trans-olecranon fracture posterior dislocation

A thorough understanding of the mechanism of injury is beneficial for treatment, especially in the treatment of elbow instability. The terrible triad is a posterolateral rotatory injury, because the forearm is axially loaded while rotating posterolaterally relative to the humerus [14]. It includes a radial head fracture, coronoid fracture, and lateral collateral ligament complex injury. Anteromedial coronoid fractures are posteromedial rotatory injuries, due to posteromedial rotation of the forearm relative to the distal humerus combined with excessive varus [24]. It includes an anteromedial coronoid fracture, disruption of the lateral collateral ligament complex, and the posterior band of the medial collateral ligament. Trans-olecranon fracture dislocations, the distal humerus is impacted across the greater sigmoid of the ulna, results in a fracture of the olecranon [6, 14]. Most of the time, once the bones are fixed, ligament repair or reconstruction is not required. Based on our experiences, when patients fall, their palm hits the floor, the forearm is rotated posterolaterally relative to the humerus, and at the same time, high energy directly hits the olecranon and results in the fracture of the olecranon. Because of the olecranon fracture, the forearm moves backward, and the humerus prevents the coronoid process and results in a fracture, and the distal humerus also shears off and fractures the radial head. Also, elbow dislocation occurs if the degree of rotation is large enough. During the process of treatment, we found that even if the bony structure is restored, the elbow joint is also dislocated. Elbow joint stability is restored after the lateral collateral ligament complex is repaired.

### Treatment of trans-olecranon fracture posterior dislocation

Obviously, trans-olecranon fracture posterior dislocation is an unstable injury of the elbow joint and operation is necessary. The three bony articulations of the elbow (ulnohumeral, radiocapitellar, and proximal radioulnar) allow for movement in the flexion–extension axis as well as forearm rotation [25]. The goal of the operation is to restore the normal anatomy of the joints. It is also conducive to start early elbow functional exercise and recovery of daily activities. Due to early surgical treatment, good anatomical reduction and firm internal fixation can further restore elbow function. The most commonly used surgical approach being the posterior median approach can be used to simultaneously perform the reduction and fixation of the fracture of the olecranon, coronoid process, and radial head. This approach has a small lesion and clear visual field, with a small neurovascular lesion, but with a limited exposure to the coronoid process. Therefore, if necessary, the anterior approach can be combined to better expose the coronoid process. In this study, all patients had the posterior median approach performed, with only one patient having an additional anterior approach.

The choice of internal fixation is also important. Trans-olecranon fracture posterior dislocation is mostly combined with a coronoid process fracture, and as the fragment of the coronoid process is large, rigid internal fixation is crucial to obtain a good outcome. Because of the fracture and displacement of the olecranon, the coronoid process can be reduced and fixed from the rear. Fixation with a hollow lag screw or Kirschner wire can obtain reliable stability, so most injuries do not need to be fixed with the forward approach and plate. Most of the fractures of the olecranon are comminuted fractures, which are extremely unstable. Anatomical reduction should be made as far as possible during the operation, and proper and firm internal fixation is necessary to achieve the basic shape and stability of the elbow joint. Villanueva [14] considered that the shape of the olecranon determines that the best configuration of fixation is plate fixation, not tension band wire fixation. Because the fixed strength of the tension band wire is not enough, it may cause the olecranon to be compressed and shortened, resulting in elbow joint instability which will affect joint movement, and finally lead to traumatic arthritis. Brink [26] believed that fixation with tension band wire has a high failure rate in patients with an unstable elbow joint. In our opinion, in comminuted olecranon fractures, especially combined with elbow dislocation, the tension band wire cannot be firmly fixed, and displacement and loosening are easy to occur. Therefore, a plate, especially the anatomical locking plate, is a better choice for the treatment of trans-olecranon fracture posterior dislocation. In this study, eight patients had an anatomical locking plate to fix the olecranon fracture. The repair of ligaments is also important to restore the stability of the elbow joint, and all patients had ligament repairs with different methods.

### Limitations

Limitations of this study include a relatively small number of patients, and it was a retrospective review. These are the inherent limitations of the study of uncommon traumatic injuries.

## Conclusions

In summary, trans-olecranon fracture posterior dislocation is a rare injury, which can result in complex elbow instability. Ligament repair is as important as bony repair to restore elbow stability. Careful study of the injury characteristics of the fracture, making operation plan, and restoring the olecranon–trochlea humeri-coronoid process anatomy is the foundation of reduction and good outcome.

## Data Availability

Please contact author for data requests.
